# Development a Recombinant Protein (CrFSH) as a Reproductive Hormone for the Assisted Reproduction of Dairy Cows

**DOI:** 10.3390/ani15101430

**Published:** 2025-05-15

**Authors:** Xinxi Qin, Haisen Zhang, Tian Liu, Zhenliang Cui, Kangkang Gao, Pengfei Lin, Yaping Jin

**Affiliations:** 1 Department of Clinical Veterinary Medicine, College of Veterinary Medicine, Northwest A&F University, Yangling 712100, China; qinxinxi@163.com (X.Q.); haisenzhang@nwafu.edu.cn (H.Z.); lt0000@nwafu.edu.cn (T.L.); zlccl2004@163.com (Z.C.); gkk@nwafu.edu.cn (K.G.); linpengfei@nwsuaf.edu.cn (P.L.); 2 Key Laboratory of Animal Biotechnology of the Ministry of Agriculture and Rural Affairs, College of Veterinary Medicine, Northwest A&F University, Yangling 712100, China

**Keywords:** FSH, GCs, CTP, cyclic adenosine monophosphate, follicular growth

## Abstract

Follicle stimulating hormone (FSH) stands as one of the most prevalently used reproductive hormones in the field of animal-assisted reproduction. Conventionally, pituitary FSH is sourced from the heterologous pituitary glands of pigs and sheep procured from slaughterhouses, and it typically exists in the form of crude FSH. The specific challenges inherent in FSH-based assisted reproduction drugs has significantly spurred the interest in exploring novel alternatives, aiming to reduce the reliance on these traditional sources in relevant production processes. In this study, the α- and β-FSH genes were retrieved from pituitary cDNA libraries. These genes were selected to construct a recombinant protein—the novel cow recombinant FSH (CrFSH)—through the application of the homologous recombination method. Notably, the β-subunit was extended by a carboxy-terminal peptide (CTP). After successfully integrating the two genes into Chinese hamster ovary (CHO) cells, the recombinant protein (approximately 33 kDa) in the culture supernatant was detected using Western blotting (WB). The results of the GCs proliferation experiment indicated that both 1.2 µg/mL pFSH and 20–20,000 ng/mL CrFSH could significantly promote the proliferation of GCs in vitro. Remarkably, on the 4th day after treatment, 20 ng/mL of CrFSH had a higher GCs proliferation rate than 1.2 μg/mL of pFSH (*p* < 0.001). Additionally, cyclic adenosine monophosphate (cAMP) induction assay in GCs unequivocally confirmed that CrFSH possesses superior activity compared to pFSH. These findings underscore that this recombinant protein holds great potential as a promising candidate for FSH production in assisted reproduction approaches for dairy herds.

## Simple Summary

This study aims to obtain biologically active recombinant long-acting bovine follicle-stimulating hormone (CrFSH) through gene recombination technology. Initially, the complete coding sequence (CDS) of bovine FSH was cloned, followed by the addition of a CTP sequence to the C-terminus of the β-subunit. Subsequently, a lentiviral vector was employed to successfully integrate the recombinant gene into the genome of CHO-K1 cells, and the presence of CrFSH protein was detected in the cell supernatant. Finally, the impacts of CrFSH and pituitary-derived FSH (pFSH) on granulosa cell (GC) proliferation rates, cyclic adenosine monophosphate (cAMP) induction, and the expression of target genes within GCs were compared. The experimental findings revealed that CrFSH not only significantly enhanced GC proliferation and effectively regulated the expression of target genes but also substantially increased the intracellular cAMP content. Collectively, these results provide compelling evidence that CrFSH outperforms pFSH in terms of biological activity, suggesting its promising potential as a novel FSH-based pharmaceutical agent.

## 1. Introduction

Globally, the utilization of in vitro embryo production (IVP) and in vivo derived (IVD) technologies has boosted the application of sexed semen (SS) in the production of replacement females or males within the realm of dairy breeding. Data from the International Embryo Technology Society (IETS) reveal that the dominant trends in the global embryo industry have been consistently on an upward trajectory. This is substantiated by the annual report which details the embryo transfer (ET) activities collected across the world from 2003 to 2022. Moreover, although the embryo industry endured short-term impacts due to the pandemic between 2021 and 2023 [1], such as shutdowns and disruptions to logistics and supply chains, the economic and political landscapes differed from one country to another. Nevertheless, ET has superiority over natural reproduction in terms of improving the rate of genetic improvement [2,3,4].

In Northern China, pastures are home to approximately 90% of the country’s dairy cattle population. Historically, these herds were often managed using traditional extensive practices, leading to consistently low individual milk production across these northern regions and throughout the rest of the country. By 2024, milk production in the 10 northern provinces of China had been projected to constitute around 83% of the national total. However, with an annual milk yield of less than 10 tons per cow on average, this situation poses a significant constraint on the efficient utilization of the country’s cattle breeding resources [5]. To tackle these problems, ET has emerged as a crucial reproductive tool capable of transforming the production systems for individuals possessing high genetic merit and has the potential to enhance dairy performance [6,7]. In fact, ET has been documented in China for a minimum of 40 years. Although some progress has been made in the synchronization of estrous cycles and ovarian super-stimulation promoted by reproductive hormones, there has been no significant improvement in the average number of embryos produced by ovarian super-stimulation or the successful pregnancy rate per ET in Chinese dairy farms [8,9].

In recent years in China, the breeding of dairy cows has been founded on the application of imported breeding livestock and embryos. This involves the use of pregnant mare serum gonadotropin (PMSG), which is purified from the blood of pregnant mares during the period between 35 and 100 days of gestation, or FSH sourced from porcine and ovine pituitary glands obtained from slaughterhouses [10]. Such reliance on traditional methods demands a well-established operational system and strict biosafety measures. Compared to PMSG extracted from the serum of pregnant mares, CrFSH produced entirely in vitro does not raise animal welfare concerns and is not limited by the availability of raw materials. Based on investigations and the ongoing serious concerns raised by the production and use of PMSG in the EU, Eurogroup for Animals urges the EU to introduce a full ban on the production and use of PMSG and revise the animal welfare legislation [11,12]. Additionally, it should be emphasized that these two types of products may possess either FSH or LH activity and are used to induce superovulation in cow. However, our objective is to generate only FSH activity to acquire more high-quality cow follicles for breeding purposes [13,14,15].

In the present study, the lentiviral overexpression vector of the CrFSH gene was first constructed. Then, after transfecting CHO cells, a high-yield CrFSH recombinant protein containing the FSHα-subunit and the β-subunit with one CTP was obtained, and its biological activity was identified. The CrFSH fusion protein expressed in CHO cells was generated using genetic engineering techniques. We hypothesized that CrFSH has high biological activity. To this end, we evaluated the biological activity of CrFSH through in vitro assays. The data showed that GC proliferation, cAMP induction assay, and key genes related to follicle development exhibited high FSH biological activity. Collectively, these data support the potential utility of CrFSH in reproductive biotechnology applications, where controlled modulation of follicular development is critical. In vitro assays provide a robust framework for evaluating its biological activity. Moreover, the recombinant expressed protein has been proven to be superior to the crude tissue-derived FSH in terms of biosafety [16,17,18]. Ultimately, this approach will offer better control over the production source, ensure greater consistency and reproducibility from batch to batch and eliminate any bioethical concerns and animal welfare issues.

## 2. Materials and Methods

### 2.1. Cells and Vectors

The Chinese hamster ovary cells (CHO-K1) were kindly provided by Stem Cell Bank, Chinese Academy of Sciences (Shanghai, China) [19]. The 293T cells and pcDNA3.1(+) vector were previously retained in our laboratory. The lentiviral vector systems, namely pCDH-CMV-MCS-EF1-GFP (pCD513B), pRSV-Rev, pMD2.G, and pMDLg/pRRE, were also maintained within our laboratory. GCs were isolated from ovaries of Holstein dairy cows and previously cryopreserved in our laboratory under standardized conditions.

### 2.2. Design and Construction of the CrFSH Gene

We completed the assembly of plasmids expressing the chimera hormone gene with one CTP. Briefly, the CrFSH, which encompasses the FSH-CTP chimera, was fabricated by splicing the *Bos taurus* FSHβ gene from the carboxyl-terminus of hCG up to the termination codon and then integrating it with the *Bos taurus* FSHα gene. Subsequently, the synthetic sequences of FSHα-subunit and FSHβ-CTP cDNA, flanked by *Bam*H I (3′) and *Hind* III (5′) sites, were inserted into a pcDNA3.1(+) vector, generating pcDNA3.1-CrFSHα and pcDNA3.1-CrFSHβ-CTP, respectively.

**Figure 1:**
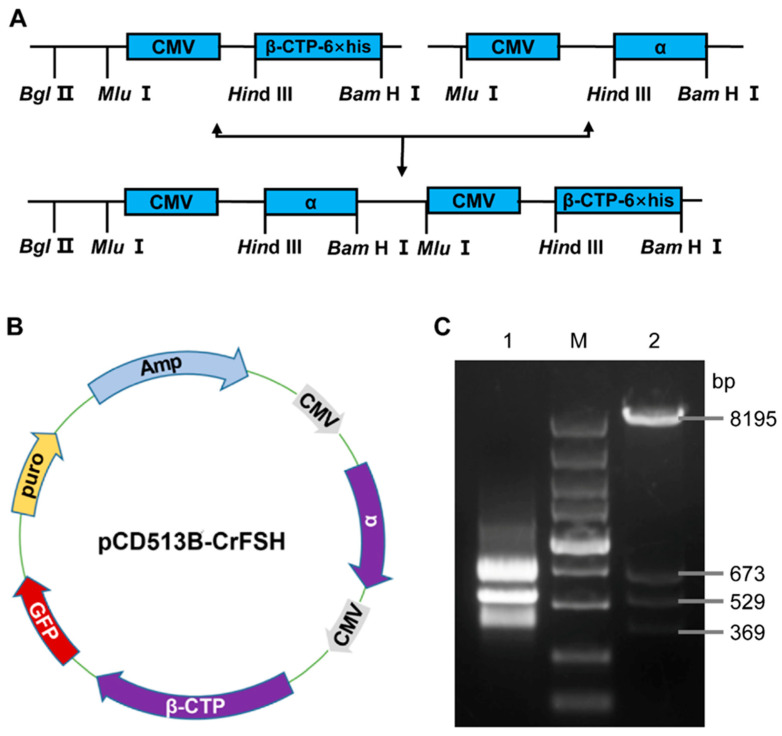
Construction and identification of the pCD513B- CrFSH. (**A**) Construction flowchart of CrFSH recombinant gene; (**B**) schematic diagram of the pCD513B-CrFSH vector, which included two target fragments, CMV-FSHα and CMV-FSHβ-CTP-6×His; (**C**) identification map of CrFSH recombinant gene. M indicates the DL5000 DNA marker; 1 indicates the fragment of FSHα-CMV-β-CTP-6×His; 2 indicates the pCD513B-CrFSH vector (β-CTP-6×His: 529 bp; CMV: 673 bp; α: 369 bp).

A 1046 bp CrFSHα fragment, digested with *Mlu* I-*Hin*d III from pcDNA3.1-CrFSHα, was incorporated into pcDNA3.1-CrFSHβ-CTP that was digested with *Mlu* I prior to ligation, thus generating pcDNA3.1-CrFSH. The CrFSHβ-CTP gene was amplified via PCR with a 6 × His-tag at the 5′ terminus and was then sub-cloned into a pcDNA3.1 vector. The resultant pcDNA3.1-CrFSH plasmid harbored two cytomegalovirus promoters and genes encoding for BFSHα and CrFSHβ-CTP. After purifying the pcDNA3.1-CrFSH plasmid, we verified the expression of the two fragments through *Hin*d III and *Bam* H I digestion followed by agarose gel electrophoresis analysis. Subsequently, an approximately 1.6 kb amplified sequence was integrated into the lentiviral vector (pCD513B-CrFSH) via genetic recombination, which was also analyzed using agarose gel electrophoresis. The schematic diagram for plasmid construction is illustrated in Figure 1 and Supplementary Table S1.

A 1046 bp BFSHα fragment, digested with *Mlu* I-*Hin*d III from pcDNA3.1-CrFSHα, was incorporated into pcDNA3.1-CrFSHβ-CTP that was digested with *Mlu* I prior to ligation, thus generating pcDNA3.1-CrFSH. The CrFSHβ-CTP gene was amplified via PCR with a 6 × His-tag at the 5′ terminus and was then sub-cloned into a pcDNA3.1 vector. The resultant pcDNA3.1-CrFSH plasmid harbored two cytomegalovirus promoters and genes encoding for BFSHα and CrFSHβ-CTP. After purifying the pcDNA3.1-CrFSH plasmid, we verified the expression of the two fragments through *Hin*d III and *Bam* H I digestion followed by agarose gel electrophoresis analysis. Subsequently, an approximately 1.6 kb amplified sequence was integrated into the lentiviral vector (pCD513B-CrFSH) via genetic recombination, which was also analyzed using agarose gel electrophoresis. The schematic diagram for plasmid construction is illustrated in Figure 1 (Supplementary Table S1).

### 2.3. The Lentiviral Vector Transfection

The recombinant lentiviral plasmid (pCD513B-CrFSH) expressing green fluorescent protein (GFP) was co-transfected with virus packaging plasmids (pRSV-Rev, pMD2.G and pMDLg/pRRE) into 293T packaging cells using Lipo8000™ (Beyotime, #C0533, Shanghai, China) when the cells reached 70–80% confluence. After overnight transfection, the medium was replaced, and the cell supernatants containing the virus were harvested at 48 h and 72 h post-transfection. The viral culture medium was filtered through a 0.22 μm filter. Subsequently, Polybrene (Solarbio, Beijing, China, NO: H8761) was added to a final concentration of 8 μg/mL. Then, the resulting medium was used to transfect CHO-K1 cells. After 24 h, the transfected cells were allowed to recuperate by replacing the medium and were selected with Puromycin Dihydrochloride (Beyotime, #ST551) at 3 μg/mL for the initial less stringent selection. Subsequently, concentrations of 5, 8, and 10 μg/mL were used for the following three passages to conduct a more stringent selection.

### 2.4. Monoclonal Cell Isolation and Identification

The limiting dilution technique was employed to isolate individual clones that were transformed by the lentiviral vector and carried both the CrFSH genes and the GFP mRNA. The successful transcription was observable as detectable green fluorescence using a Zeiss Axio Observer Z1 microscope (Zeiss Microscopy, Oberkochen, Germany). In this approach, theoretically, approximately half of the 96-well plates contained a single cell at a concentration of 0.5 cells/100 μL. Likewise, the expanded monoclonal cells needed to be stored for further analysis. Motivated by the imaging outcome, reverse transcription polymerase chain reaction (RT-PCR) was used to identify the DNA extracted from the expanded monoclonal cell populations. Upon evaluating the quality of the PCR products via gel electrophoresis, the sequence was ascertained by Tsingke Biotech Co., Ltd., Xi’an, China, further validating the presence of the target genes.

### 2.5. Western Blot Analysis

To detect the secreted recombinant protein, total proteins were extracted from the cell supernatants centrifuged at 6000× *g* for 10 min. Before electrophoresis, protein concentration was assessed with NanoDrop 2000 (Thermo Scientific, Wilmington, DE, USA), and then, the samples were heated to 100 °C for 5 min and cooled on ice immediately. Equivalent amounts of proteins were separated by 12.5% SDS–PAGE gel electrophoresis, electro-transferred onto PVDF membranes, sealed with 5% skimmed milk powder for 2 h and incubated overnight at 4 °C with anti-FSHα (1:500; DF6371, Affinity Biosciences, Shanghai, China) or anti-His-tag (1:1000; Proteintech Group, Inc., Wuhan, China). After washing with TBST (TBS containing 1% Tween), the membranes were incubated with a secondary antibody (conjugated to horseradish peroxidase, HRP, Proteintech) at ambient temperature for 1 h, and protein levels were detected using the ECL protein blotting detection kit (Kejie Biotechology Inc., Shanghai, China).

### 2.6. Purification of CrFSH

In brief, collected supernatants was filtered through a 0.45 μm filter (Tianjin Jinteng Experimental Equipment Co., Ltd., Tianjin, China) and concentrated ten-fold by ultrafiltration using the 50000 and 5000 NMWC Hollow Fiber Cartridges (Cytiva: UFP-50-C-3MA, Global Life Sciences Solutions USA LLC., Marlborough, MA, USA). Finally, the concentration of the purified fusion protein was detected by NanoDrop One (Thermo Fisher, Waltham, MA, USA).

### 2.7. The Cell Proliferation Rate Was Detected by the CCK-8 Method

pFSH (Ningbo Second Hormone Factory, Ningbo, China) was dissolved in a culture medium (DMEM/F-12 + 10% (*v*/*v*) fetal bovine serum (FBS) + 1% (*v*/*v*) penicillin–streptomycin solution (PS)) to prepare a 12 mg/mL solution and then sterilized through a 0.22 μm filter. Subsequently, the PS was used to dilute pFSH to different concentrations: 0 ng/mL, 12 ng/mL, 120 ng/mL, 1.2 μg/mL, 12 μg/mL, and 120 μg/mL. Similarly, CrFSH was prepared into different concentrations: 0 ng/mL, 2 ng/mL, 20 ng/mL, 200 ng/mL, 2 μg/mL, and 20 μg/mL. Meanwhile, GCs at a density of 2 × 10^4^/mL were seeded at 100 μL per well into 96 well plates and treated with different concentrations of pFSH and CrFSH. Each concentration had six replicate wells and was cultured for 1 d, 2 d, 3 d, 4 d, 5 d, 6 d, and 7 d. At different time points, 10 μL of the CCK-8 reagent (Mishu (Xi’an) Biotechnology Co., Ltd., Xi’an, China) was added to each well to detect cell viability. Two hours later, the absorbance at 450 nm was determined via a microplate reader (Bio-Rad, Hercules, CA, USA)

### 2.8. cAMP-Based Enzyme Immunoassay

GCs were employed to assess the cAMP production in response to the stimulation of pFSH and CrFSH. The levels of cAMP were quantified using a Bovine cAMP Enzyme-Linked Immunosorbent Assay (ELISA) kit (Jiangsu Meimian Industrial Co., Ltd., Yancheng, China) in strict accordance with the manufacturer’s instructions. Intra-assay and inter-assay coefficients of variation (CVs) were established as <10% and <15%, respectively.

In 96-well plates, 100 μL of the culture medium containing GCs (2 × 10^4^ cells/mL) was added to each well. After 24 h of incubation, the cells were further cultured for 1 h at 37 °C in the medium with 1.2 μg/mL of pFSH and 20 ng/mL and 200 ng/mL of CrFSH separately. Subsequently, the cells were lysed to measure the cAMP levels.

### 2.9. RNA Isolation and qRT-PCR

GCs were cultured in 6-well plates using a complete DMEM/F12 medium. Following overnight culture, the culture medium was replaced with a fresh medium after the addition of 1.2 μg/mL of pFSH and 20 ng/mL of CrFSH. After 24 h treatment, the GCs were harvested for RNA extraction and qRT-PCR analysis. RNA was extracted using RNAiso Plus (TaKaRa, Tokyo, Japan) and then converted into cDNA with the Evo M-MLV reverse transcription kit (containing gDNA removal reagent for qPCR) (Hunan Aikerui Bioengineering Co., Ltd., Changsha, China).

Subsequently, qRT-PCR was carried out to analyze the expressions of key genes, including *Fshr*, *Lhcgr*, and *Cyp19a1*. The 2×Fast qPCR Master Mixture (Beijing Dinning Biotechnology Co., Ltd., Beijing, China) was used for this purpose. Each qPCR reaction was performed in triplicate. The primers employed in the experiment are listed in Supplementary Table S2. The expression of β-actin was used as a reference gene for each sample.

### 2.10. Statistical Analyses

A Shapiro−Wilk test was performed to assess the normal distribution of the data. All data are presented as the mean ± standard deviation (SD). A paired *t*-test was employed to analyze the differences between groups. GraphPad Prism 8.0 (GraphPad, located in San Diego, CA, USA) was used for the generation of graphical representations. Statistical analysis for data included a paired *t*-test for paired data comparison between two groups and ANOVA with Tukey’s or Sidak’s test for data comparison between multiple groups. Statistical significance was defined as *p* < 0.05.

## 3. Results

### 3.1. Construction and Identification of the rBFSH Expression Lentiviral Vector

The construction flow chart of the CrFSH recombinant gene is shown in Figure 1A. The FSHα-subunit and FSHβ-CTP genes were cloned into a lentiviral vector (pCD513B-CrFSH), containing 6×His-tag. The map of the expression vector was produced by SnapGene and visualized using ChimeraX (Figure 1B). The schematic of CrFSH genes for plasmid construction is shown in Figure 1A (Figure 1A presents a schematic diagram of the CrFSH genes used for plasmid construction), and the mapped recombinant protein placed in the model structure consisting of the α-subunit and the β-CTP-6×His subunit was visualized using ChimeraX (Figure 1B). Figure 1C showed the results of a 1.6 kb FSHα-CMV-β-CTP fragment and a lentiviral vector were digested by *Bam* H I and *Nhe* I.

### 3.2. Isolate the Genes of CrFSH-Carrying Monoclonal by the Limiting Dilution Method and Identify CrFSH

**Figure 2:**
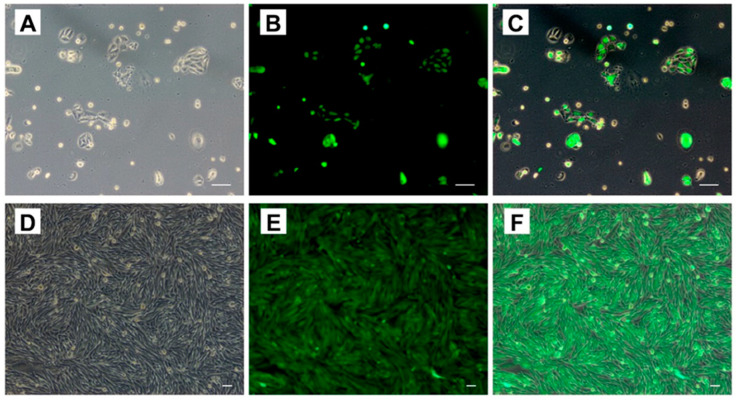
Establishment of a stable cell line expressing CrFSH. (**A**–**C**) Polyclonal CHO-K1 cell lines; (**D**–**F**) monoclonal CHO-K1 cell lines obtained through three rounds of screening. (**A**,**D**) represent bright-field images of CHO-K1 cells, whereas the green fluorescent signals in (**B**,**E**) indicate successful transfection of recombinant lentivirus and expression of GFP in CHO-K1 cells.

**Figure 3:**
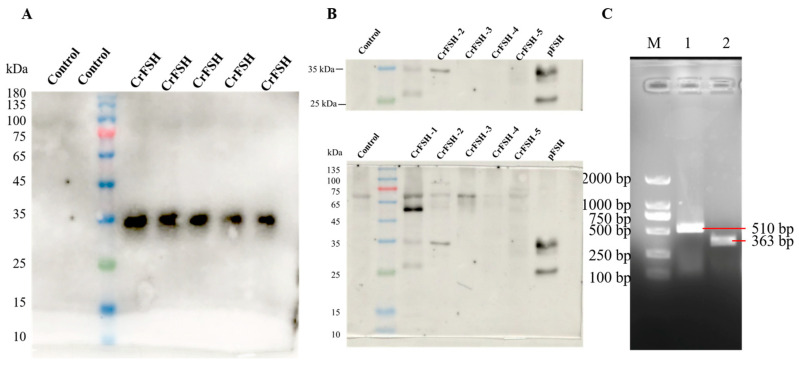
Purification and identification of CrFSH. (**A**) Identification the supernatant of CrFSH by Western blot with anti-His-tag (control: negative control supernatant; five samples of CrFSH: supernatant of monoclonal cell lines). (**B**) Identification the purification of the CrFSH supernatant by Western blot with anti-FSHα. It indicates that there is an α-subunit with a structure similar to the commercial pFSH (Ningbo Second Hormone Factory, Ningbo, China) in the supernatant. (**C**) Identification of the FSHβ-CTP-6×His gene and FSHα gene in the monoclonal cell line (M indicates DL2000 DNA marker; 1 indicates the FSHβ-CTP-6×His gene (510 bp); 2 indicates the FSHα gene (363 bp)).

By making use of the polyclonal cell strain which had the ability to detect the His-tag in the supernatant, monoclonal cell strains were isolated via the limiting dilution method (Figure 2). Eventually, five monoclonal cell strains, named 4201−4205, were successfully obtained and then expansion-cultured. The secreted proteins in the supernatant of the monoclonal cells were probed with an FSHα antibody. After two rounds of WB analysis, it was clearly shown that the CrFSH-2 protein was present at around 35 kDa (Figure 3A,B). RT-PCR was used for the identification process, and it was also verified that the FSHα-subunit and FSHβ-CTP genes were stably incorporated into the sub-cultured monoclonal cell strains (Figure 3C).

### 3.3. The GCs Proliferation Rate

**Figure 4:**
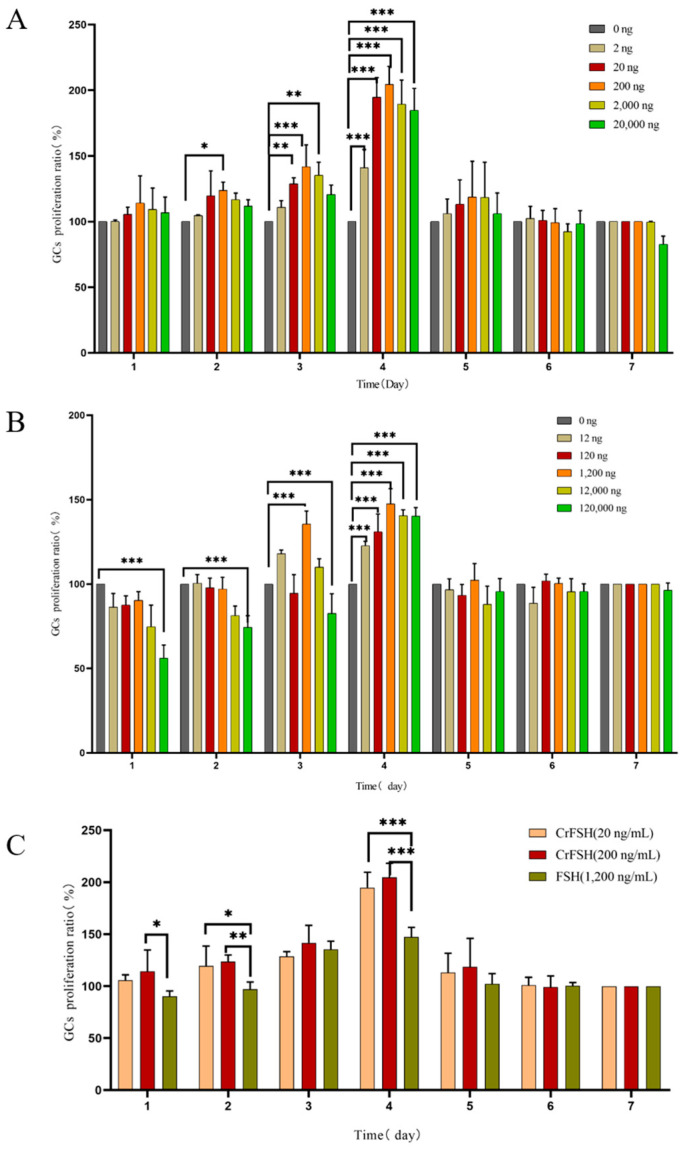
The effects of pFSH and CrFSH on the proliferation rate of GCs were detected by CCK-8 assay. (**A**) The cell proliferation rate reached its peak on the 4th day of pFSH treatment. At this time point, all concentrations of pFSH could significantly increase the proliferation rate of GCs, but high concentrations of pFSH could inhibit the proliferation of GCs. (**B**) The cell proliferation rate reached the peak at the 4th day of CrFSH treatment. The time-dose-effect trend of CrFSH on the proliferation rate of GCs was similar to that of pFSH but showed a better promotion effect. (**C**) Comparison of the optimal doses of CrFSH and pFSH on the proliferation rate of GCs. The effects of 20 ng/mL and 0.2 μg/mL of CrFSH on the proliferation rate of GCs were significantly higher than 1.2 μg/mL of pFSH (*, *p* < 0.05; **, *p* < 0.01; ***, *p* < 0.001).

The results indicated that as the treatment time extended, the proliferation rate of GCs reached its peak on the 3rd and 4th days of culture (Figure 4A). By comparing the effects of different concentrations of FSH and CrFSH on the cell proliferation rate, it was found that 1.2 μg/mL of FSH, along with 20 ng/mL and 200 ng/mL of CrFSH, significantly enhanced the proliferation rate of GCs. The differences were statistically significant compared with other concentrations (*p* < 0.05) (Figure 4B). Additionally, the effects of 20 ng/mL and 200 ng/mL of CrFSH on the proliferation rate of GCs were significantly higher than those of 1.2 μg/mL of FSH (*p* < 0.05) (Figure 4C). Based on these results, the optimal dose for the cAMP experiment was determined.

### 3.4. cAMP Induction Assay

**Figure 5:**
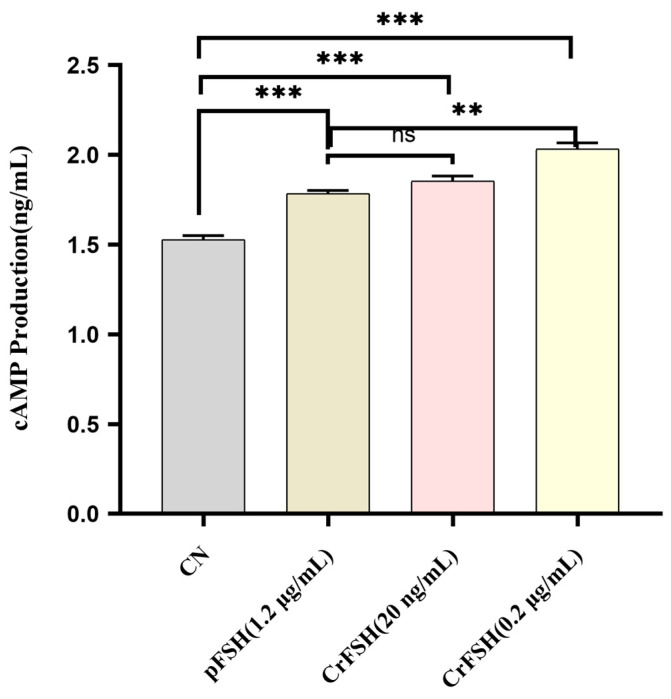
Comparison of cAMP production induced by 1.2 μg/mL of pFSH and 20 ng/mL and 200 ng/mL of CrFSH in vitro. ns, no significant; **, *p* < 0.01; ***, *p* < 0.001.

The FSH receptor (FSHR) is a member of the G-protein-coupled receptor (GPCR) family [20]. The interaction of FSH with the FSHR on GCs initiates the synthesis of cAMP [21]. The results of induction assay showed that, 1 h following the individual addition of pFSH and CrFSH to GCs, the cAMP levels in the treatment groups were significantly higher than those in the control group (*p* < 0.05). However, under treatment conditions of 1.2 μg/mL pFSH and 20 ng/mL CrFSH, no statistically significant difference in cAMP content within GCs was observed (*p* > 0.05) (Figure 5). Notably, consistent with the CCK-8 cell proliferation rate assay, 200 ng/mL CrFSH showed a higher cAMP-induced yield compared with other CrFSH dose groups and pFSH-treated groups.

### 3.5. The Genes Expression in GCs Treated with pFSH and CrFSH

**Figure 6:**
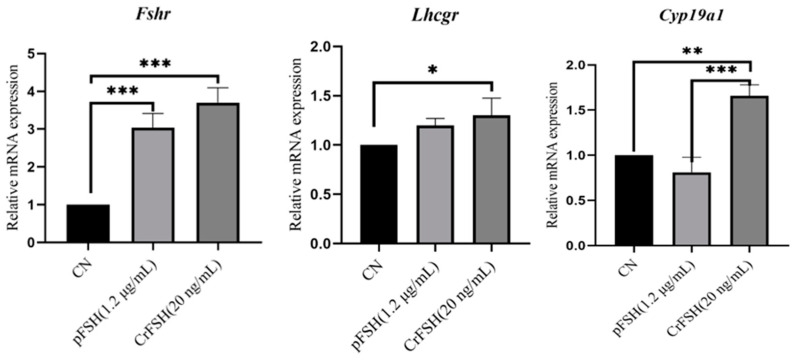
Gene expression analysis in GCs. RT-qPCR was employed to evaluate follicular development-associated gene expression (*Fshr*, *Lhcgr*, and *Cyp19a1*) in GCs following pFSH or CrFSH treatment. Transcript levels were normalized to β-actin mRNA, with the control group expression baseline established as 1. The results showed the relative expression levels by one-way ANOVA. *, *p* < 0.05; **, *p* < 0.01; ***, *p* < 0.001.

Following a 24 h treatment period, qRT-PCR analysis was conducted to quantify the expression levels of key follicular development-associated genes (*Fshr*, *Lhcgr*, and *Cyp19a1*). Both treatment groups exhibited significant upregulation in *Fshr* and *Lhcgr* mRNA expression compared to the control group (*p* < 0.05). Notably, the CrFSH group demonstrated elevated *Cyp19a1* transcript levels relative to both the control and pFSH-treated groups (*p* < 0.05). No statistically significant difference in *Cyp19a1* expression was observed between the FSH-treated group and the control group (*p* > 0.05) (Figure 6).

## 4. Discussion

The FSH protein is a heterodimer composed of α- and β-subunits [22]. The α-subunit is common to other pituitary glycoprotein hormones, including luteinizing hormone (LH), thyroid-stimulating hormone (TSH), and placenta derived human chorionic gonadotropin (hCG). In contrast, the β-subunit is unique and endows the hormone with biological specificity. Subunit assembly is crucial not only for the bioactivity of FSH [23] but also for the stability of the β-subunit. With the development and popularization of recombinant gene technology and high-precision cell culture technology since the 20th century, researchers have successfully constructed various types of recombinant FSH (rFSH) in vitro. Evidence indicates that a single-chain fusion of the α- and β-subunits of FSH is fully active. When fused with the carboxy-terminal peptide of hCG, it can significantly extend the serum half-life [24,25]. Moreover, studies suggest that glycosylation modification or the addition of protective fragments to rFSH can enhance its in vivo stability and efficacy.

Based on these findings, we designed and constructed FSH analogs to improve their survival in the circulatory system. The CrFSH is a non-covalent heterodimer consisting of an α-subunit and a β-subunit with a CTP attached to the carboxy terminus, which offers a more convenient dosing schedule for clinical use [26]. The rhFSH-CTP has an elimination half-life 2–4 times longer than that of FSH [27], likely due to the four closely spaced O-oligosaccharides in the CTP that are assumed to delay hormone metabolism in vivo [28].

To ensure the accuracy of our constructs, we confirmed the precision of the two-chain molecule and the lentiviral vector by using PCR, RT-PCR, and sequencing. Additionally, Western blot (WB) analysis demonstrated a prominent band at around 35 kDa, which was concurrently recognized by both the α antibody and the 6×His-Tag antibody. This band corresponded precisely to the predicted molecular weight of the CrFSH. Subsequent assays verified that both this band and pFSH could be identified by the α antibody. These results further suggest that the CrFSH in the cell supernatant aligns well with what we anticipated in the experiment.

Moreover, we conducted further investigations into the biological activity of CrFSH. Given that pFSH mainly acts on GCs of the follicles, early GCs show a significant dose-dependent response to FSH [23,25,29,30,31]. pFSH plays a key role in follicular survival because it stimulates the proliferation and differentiation of GCs and indirectly participates in regulating the meiosis of oocytes [32,33,34]. The proliferation rate of GCs peaked at 1.2 μg/mL of pFSH and 200 ng/mL of CrFSH. These findings suggest that an appropriate dosage of pFSH significantly influences cell proliferation, apoptosis, and follicle formation. Previous research has demonstrated that in poultry, treatment with FSH upregulates genes associated with cell proliferation or apoptosis, thereby stimulating GC proliferation and inhibiting apoptosis [35,36]. Consistent with these findings, our study also observed a dose-dependent effect at low doses of pFSH and CrFSH, while a suppressive effect was evident at high doses. It is worth noting that in the above experiments, the 20 ng/mL of CrFSH showed a better effect than pFSH at 1.2 μg/mL (*p* < 0.05).

The canonical signaling pathway of FSH involves the FSHR, a GPCR that triggers the synthesis of cAMP [20,37,38]. Subsequently, we carried out a cAMP induction assay related to FSH. When FSH binds to the FSHR, which is exclusively expressed on the surface of GCs, it activates adenylate cyclase, leading to an upsurge in cAMP production [39,40]. Our findings indicated that both FSH and CrFSH are capable of inducing cAMP synthesis in GCs, but low-dose CrFSH has a higher efficacy. Evidently, these results confirm that CrFSH exhibits greater biological activity even when administered at a reduced dosage.

FSH binding to its cognate receptor FSHR in GCs initiates a cAMP/PKA signaling cascade that orchestrates spatiotemporal regulation of *Fshr*, *Lhcgr*, and *Cyp19a1* gene expression. Upon ligand−receptor engagement, activation of adenylyl cyclase elevates intracellular cAMP levels, triggering PKA-mediated phosphorylation of CREB transcription factors. Phosphorylated CREB translocates to the nucleus and binds cAMP response elements (CREs) within the FSHR promoter, thereby upregulating *Fshr* expression in a positive autoregulatory loop. Concurrently, the cAMP/PKA axis activates transcription factors including CREB and GATA4, which cooperatively bind regulatory elements in the LHCGR promoter to enhance its transcriptional output. Emerging evidence indicates that FSH may also induce paracrine signaling via epidermal growth factor (EGF)-like ligands, which could further potentiate *Lhcgr* expression through transactivation mechanisms [41,42].

For *Cyp19a1* regulation, activated PKA phosphorylates steroidogenic factor 1 (SF-1), enabling its interaction with CREB and recruitment of transcriptional coactivators to the *Cyp19a1* promoter [43]. This combinatorial action is potentiated by accessory factors such as DAX-1 and β-catenin (via Wnt/β-catenin signaling), which modulate chromatin accessibility at the *Cyp19a1* locus. A critical feedback mechanism arises from the enzymatic product of aromatase encoded by *Cyp19a1*, which converts androgens to estrogens. These estrogens bind estrogen receptors (ERs), driving ER-mediated transactivation of *Cyp19a1* and creating a feedforward loop that amplifies estrogen biosynthesis. Temporal dynamics of FSH exposure appear crucial: acute stimulation reinforces receptor expression through these positive feedback mechanisms, whereas prolonged FSH signaling may initiate desensitization pathways, including receptor downregulation and/or attenuation of downstream signaling components.

In the study, transcript levels of *Fshr*, *Lhcgr*, and *Cyp19a1* were significantly upregulated in GCs treated with CrFSH, whereas Cyp19a1 expression was downregulated in cells exposed to pFSH. These divergent responses suggest that the biological activity of CrFSH may be attributed to its unique capacity to initiate FSH-dependent signaling cascades. Specifically, FSH engagement of FSHR activates the cAMP/PKA pathway, which typically enhances *Cyp19a1* transcription through SF-1-mediated promoter activation. However, this process requires androgen substrate availability, as aromatase (*Cyp19a1*) catalyzes androgen-to-estrogen conversion. Notably, the culture medium used in this study lacked androstenedione supplementation, potentially limiting substrate availability for estrogen biosynthesis. Prolonged pFSH exposure under these conditions may therefore lead to Cyp19a1 downregulation due to substrate deprivation and/or negative feedback

According to reports, FSH drives follicle maturation and oocyte production, while LH triggers ovulation. In cow breeding, assisted reproductive technologies (ARTs) have significantly enhanced breeding efficiency, enabling producers to selectively breed livestock with desirable traits like higher milk yield, improved meat quality, and disease resistance. Therefore, it is strictly required that the hormone products used should not have LH-like effects [44]. CrFSH demonstrates batch-to-batch consistency and defined specific activity, addressing critical limitations of conventional pituitary-derived gonadotropins and PMSG such as high inter-batch variability and potential disease factors. This study primarily focuses on the acquisition of CrFSH and the functional validation of its activity at the cellular level, which also represents a limitation of the present work. Nonetheless, the ultimate goal of developing CrFSH is to facilitate the collection of a greater number of healthy oocytes in livestock breeding, which has the potential to yield substantial economic benefits.

## 5. Conclusions

Collectively, the findings presented herein provide compelling evidence that CrFSH exhibits superior efficacy in regulating follicular development and maturation compared to pFSH. Notably, the production process of CrFSH can be streamlined through straightforward optimization strategies, thereby enabling the scalable industrial manufacturing of safe, high-quality FSH. These results underscore the potential of CrFSH as a viable alternative to commercially available pFSH products.

## Data Availability

All data are contained within the article and Supplementary Materials.

## Supplementary Materials

The following supporting information can be downloaded at: https://www.mdpi.com/article/10.3390/ani15101430/s1. Table S1: The primer sequences for constructing the vectors (pcDNA3.1-FSHα, pcDNA3.1-FSHβ, and pCD513B-rFSH), along with coding sequences (CDSs) and deduced amino acid sequences of FSHα/β and CTP genes, are all presented in Supplementary Table S1. Table S2: The original images are presented in Supplementary Table S2.
