# Supplementary material for: Development a Recombinant Protein (CrFSH) as a Reproductive Hormone for the Assisted Reproduction of Dairy Cows
Source: Animals (Basel). 2025 May 15;15(10):1430. doi: 10.3390/ani15101430 (PMC12108188; doi:10.3390/ani15101430)
Supplement: Supplementary file 1 [file animals-15-01430-s001.zip › supplementary 1.pdf]

1. Supplementary

Table 1 Primer sequences for the construction of pcDNA3.1-FSHα, pcDNA3.1-FSHβ, and pCD513B-rFSH vectors.

Plasmid Vector	Primer（5’- 3’）
pcDNA3.1-FSHα	F: GCTGGCTAGCGTTTAAACTTAAGCTTATGGATTACTACAG
	R: CACTGGACTAGTGGATCCTTAGGATTTGTGATA
pcDNA3.1-FSHβ	F: GCTGGCTAGCGTTTAAACTTAAGCTTATGAAGTCTGTCCAG
	R: GACTAGTGGATCCTTAGTGGTGATGGTGATGATGTTGTGGGAGGA
pCD513B-rFSH	F: GCTAGCGAATTCATGGATTACTACAG
	R: CGCGGATCCTTAGTGGTGATGGTGAT

2. The Coding Sequence of the FSHα gene：

ATGGATTACTACAGAAAATATGCAGCTGTCATTCTGGCCATTTTGTCTCTGTTTCTGCAA
ATTCTCCATTCCTTTCCTGATGGAGAGTTTACAATGCAGGGCTGTCCTGAATGCAAGCT
AAAAGAAAACAAATACTTCTCCAAGCCAGATGCTCCAATCTATCAGTGCATGGGGTGC
TGCTTCTCCAGGGCATACCCCACTCCAGCGAGGTCTAAGAAGACAATGTTGGTCCCCA
AGAACATCACCTCGGAAGCTACATGCTGTGTGGCCAAAGCATTTACCAAGGCCACAGT
GATGGGAAATGTCAGAGTGGAGAACCACACCGAGTGCCACTGCAGCACTTGTTATTAT
CACAAATCCTAA。

3. The Coding Sequence of the FSHβ gene：

ATGAAGTCTGTCCAGTTCTGTTTCCTTTTCTGTTGCTGGAGAGCAATCTGCTGCAGAAG
CTGCGAGCTGACCAACATCACCATCACGGTGGAGAAAGAGGAATGTGGCTTCTGCATA
AGCATCAACACCACGTGGTGTGCAGGCTACTGCTACACCCGGGACTTGGTATACAGGG
ACCCAGCAAGGCCCAATATCCAGAAAACGTGTACCTTCAAGGAGCTGGTCTACGAGAC
GGTGAAAGTGCCTGGCTGTGCTCACCATGCAGACTCCCTGTACACGTACCCAGTAGCC
ACTGAATGTCACTGCAGCAAGTGCGACAGCGACAGCACTGACTGCACCGTGAGAGGC
CTGGGGCCCAGCTACTGCTCCTTCAGGGAAATCAAAGAATAA。

4. The Coding Sequence of the CTP gene：

GATGACCCCCGCTTCCAGGACTCCTCTTCCTCAAAGGCCCCTCCCCCCAGCCTTCCAA
GCCCATCCCGACTCCCGGGGCCCTCGGACACCCCGATCCTCCCACAA

5. The amino acid sequence of the protein encoded by the FSH β-CTP gene：

MKSVQFCFLFCCWRAICCRSCELTNITITVEKEECGFCISINTTWCAGYCYTRDLVYRDPA
RPNIQKTCTFKELVYETVKVPGCAHHADSLYTYPVATECHCSKCDSDSTDCTVRGLGPSY
CSFREIKEDDPRFQDSSSSKAPPPSLPSPSRLPGPSDTPILPQHHHHHH*

6. The amino acid sequence of the protein encoded by the FSHαgene：

MDYYRKYAAVILAILSLFLQILHSFPDGEFTMQGCPECKLKENKYFSKPDAPIYQCMGCCF
SRAYPTPARSKKTMLVPKNITSEATCCVAKAFTKATVMGNVRVENHTECHCSTCYYHKS*
