# Supplementary material for: Development a Recombinant Protein (CrFSH) as a Reproductive Hormone for the Assisted Reproduction of Dairy Cows
Source: Animals (Basel). 2025 May 15;15(10):1430. doi: 10.3390/ani15101430 (PMC12108188; doi:10.3390/ani15101430)
Supplement: Supplementary file 1 [file animals-15-01430-s001.zip › supplementary 2.pdf]

1. The Figure 3A

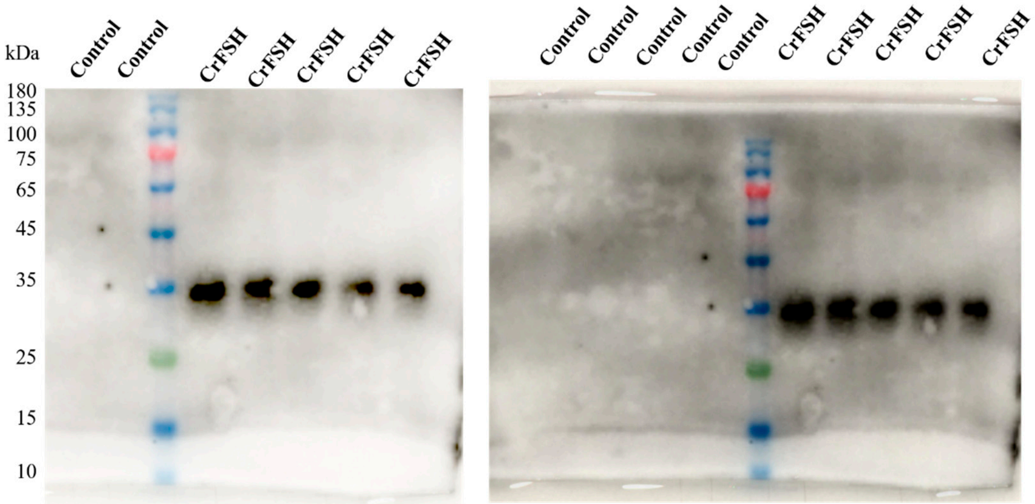

Identification the Supernatant of CrFSH by Western blot with anti-His-tag.

2. Figure 1c Agarose gel electrophoresis

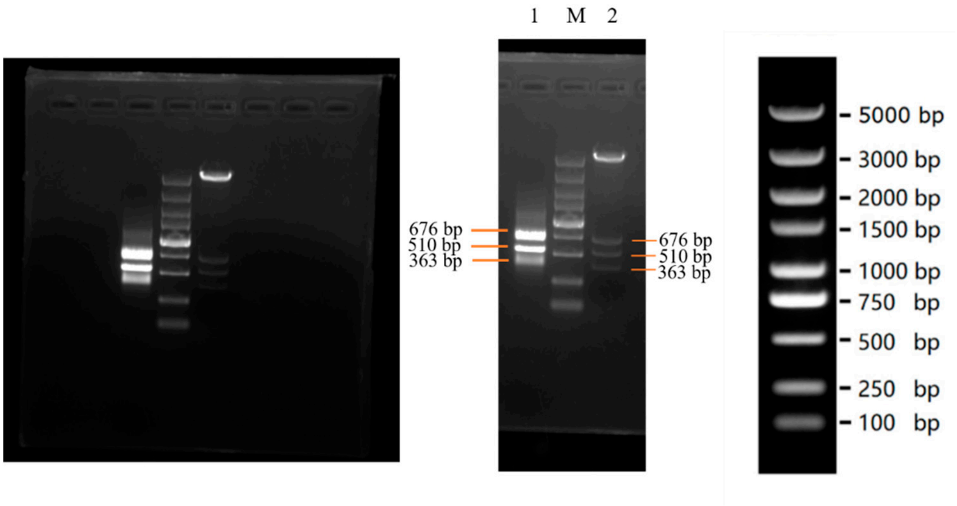

The bands shown in the agarose gel electrophoresis based on restriction enzyme cutting sites

3. Figure 3c Agarose gel electrophoresis

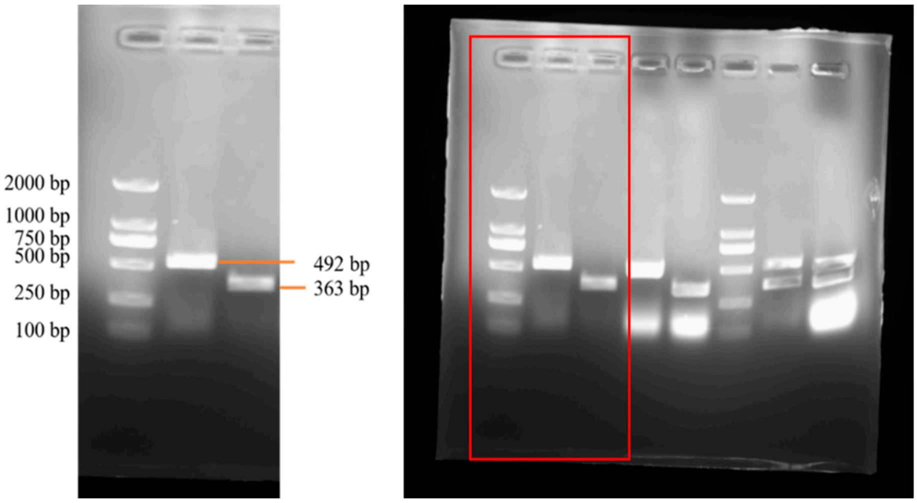

The bands shown in the agarose gel electrophoresis for PCR identification.
